# *ITPR1* variant-induced autosomal dominant hereditary spastic paraplegia in a Chinese family

**DOI:** 10.3389/fneur.2024.1365787

**Published:** 2024-07-01

**Authors:** Rui Li, Xuan Liu, Chenming Ke, Fanli Zeng, Qingyi Zeng, Xiaowei Xu, Xiaoqin Fan, Ying Zhang, Qinghua Hou

**Affiliations:** ^1^Department of Neurology, Ningbo Medical Center Lihuili Hospital, Ningbo, Zhejiang, China; ^2^Scientific Research Center, The Seventh Affiliated Hospital, Sun Yat-sen University, Shenzhen, Guangdong, China; ^3^Department of Neurology, Clinical Neuroscience Center, The Seventh Affiliated Hospital, Sun Yat-sen University, Shenzhen, Guangdong, China

**Keywords:** hereditary spastic paraplegia, *ITPR1*, whole exome sequencing, protein structure prediction, Chinese family

## Abstract

Hereditary spastic paraplegia (HSP) is a rare neurodegenerative disease prominently characterized by slowly progressive lower limb weakness and spasticity. The significant genotypic and phenotypic heterogeneity of this disease makes its accurate diagnosis challenging. In this study, we identified the NM_001168272: c.2714A > G (chr3.hg19: g.4716912A > G, N905S) variant in the *ITPR1* gene in a three-generation Chinese family with multiple individuals affected by HSP, which we believed to be associated with HSP pathogenesis. To confirm, we performed whole exome sequencing, copy number variant assays, dynamic mutation analysis of the entire family, and protein structure prediction. The variant identified in this study was in the coupling domain, and this is the first corroborated report assigning ITPR1 variants to HSP. These findings expand the clinical and genetic spectrum of HSP and provide important data for its genetic analysis and diagnosis.

## Introduction

Hereditary spastic paraplegia (HSP) consists of a group of rare genetic neurodegenerative diseases, involves the corticospinal tract, and which presents with distinct lower extremity weakness and spasticity. The estimated prevalence of HSP is 1.8–10 per 100,000 in the general population (1–4). HSP is classified based on its inheritance patterns, clinical phenotypes, and molecular pathophysiological mechanisms. It is highly heterogeneous and can be transmitted through all modes of inheritance, and includes autosomal dominant HSP (ADHSP), autosomal recessive HSP (ARHSP), X-linked HSP, and mitochondrial HSP (1). In clinical practice, HSP is classified as pure HSP when symptoms are limited to spasticity of lower limbs, bladder dysfunction, and mild somatosensory deficits, or as complex HSP when the phenotype of lower extremity spasticity is complicated by additional neurological symptoms such as macular degeneration (Kjellin syndrome) (4), positive pyramidal signs, pronounced cognitive impairment, ataxia, extrapyramidal signs, thin corpus callosum, and global brain atrophy [Mast syndrome, or spastic paraplegia (SPG21)] (5), or microcephaly, intellectual disability, and distal muscle atrophy (Troyer-like syndrome) (6).

Regarding the molecular pathophysiological mechanisms of HSPs, at least 80 different genetic loci have been associated with HSPs, and more than 60 genes have been successfully cloned (7). SPG4 is the most common type of ADHSP, accounting for approximately 40% of all cases. The main ARHSPs identified to date are SPG5, SPG7, SPG11, and SPG15 (8). However, “next-generation” sequencing studies have revealed a significant clinical and genetic overlap between different HSP subtypes and other neurodegenerative disorders such as hereditary spinocerebellar ataxias (SCAs), amyotrophic lateral sclerosis, and peripheral neuropathies (9).

In this study, we identified a HSP patient and applied direct sequencing and other genetic screening assay to unveil the mutations in the whole family. A heterozygous variant in inositol 1,4,5-triphosphate receptor type 1 (ITPR1) gene (NM_001168272: c.2714A > G, N905S) was detected in the proband and other members. Our data provided important information for genetic analysis and diagnosis of HSP.

## Materials and methods

This study was approved by the Medical Ethics Committee (KY2020PJ051) of the Ningbo Medical Center Lihuili Hospital (Ningbo, China). Prior to the study, written informed consent for genetic tests and publication of case details was obtained from all adult participants and the parents or legal guardians of the children involved in the study, and all procedures were conducted according to the Declaration of Helsinki.

### Study participants

The study participants belonged to a three-generation family with 19 members, three of whom presented with symptoms. The proband (I-2) was a 67-year-old woman, with a history of type II diabetes mellitus for 5 years. She experienced a gradual progression of gait abnormalities and ambulatory disability for approximately 20 years. Upon admission, she presented typical spastic gait, and positive pyramidal sign on both side and no significant muscle atrophy and sensory loss. Further medical history investigation indicated this lady’s deceased mother had the same spastic gait before she lost her independent walking capability. In person, physical examination confirmed one of her brothers and her son also presented symptoms and physical signs much the same as her. She was suspected of having HSP according to the Fink criteria for HSP (10), and after HSP diagnosis was confirmed by whole exome sequencing (WES), other family members were interviewed and examined; two asymptomatic family members were identified, and a pedigree study was conducted to confirm that they carried the same variant.

### Genetic screening and variant analysis

Peripheral blood samples were obtained from the patient and her family members with EDTA tubes. Comprehensive genetic testing was performed at the Oumeng V-Medical Laboratory (Hangzhou, Zhejiang, China). Total genomic DNA was extracted from peripheral blood samples using the HiPure universal DNA kit (Magen Biotechnology, Guangzhou, China, D3018), according to the manufacturer’s instructions. WES was performed on the proband and other family members, and single nucleotide variants and copy number variants were analyzed based on the sequencing data. In brief, the WES was performed by Nimblegen SeqCap EZ Exome Kit and Illumina HiSeq X Ten sequencing platform. Library preparation was performed as described in the KAPA Hyper Prep Kit (KR0961, KAPA Bio systems). In addition, dynamic mutation detection and gene microarray analysis (Affymetrix CytoScan 750 K, Thermo Fisher Scientific, United States) were performed on the proband to further confirm the results. Sanger sequencing of the PCR products was performed to validate the heterozygous variant (NM_001168272: c.2714A > G, N905S) in the inositol 1,4,5-triphosphate receptor type 1 gene, revealed by single nucleotide variant analysis, and the following primers (forward primer: CCATAAGGAAGGTCCAAGTCTG, reverse primer: GATGTGGCATCACATATAGGG) were used in PCR amplification for this purpose. Amino acid conservation of ITPR1 in different species was evaluated using the DNAMAN 9.0 software (LynnonBiosoft, United States), while the pathogenesis of the variant was evaluated using Polymorphism Phenotyping v2^1^ (11), Sorting Intolerant from Tolerant^2^ (12), and MutationTaster^3^ (13).

### Protein structure prediction

Based on the patient’s sequencing data, the protein structure was assessed using the alphafold2 software, and the scripts and commands used were as follows: python3 docker/run_docker.py --fasta_paths=/ data/ftp/lsy/alphafold/seq/$i.fasta--max_ template_date = 2022-06-14 --model_preset = monomer –data dir=/data/ alphafold2-data/ --output_dir=/data/ftp/lsy/alphafold/out/$i --gpu_devices = 1. The predicted structure of ITPR1 N905S was compared with that of ITPR1 (reference sequence: NP_001161744.1) and further docked with the electron microscopy structure of its homologous protein ITPR3, resolved using Cryo-EM (14). To identify the significance of the altered protein structure caused by the variant in the pathogenesis of SPG, the mutation was assimilated with all other ITPR1 variants recognized to be associated with SPGs (15, 16).

## Results

**Figure 1:**
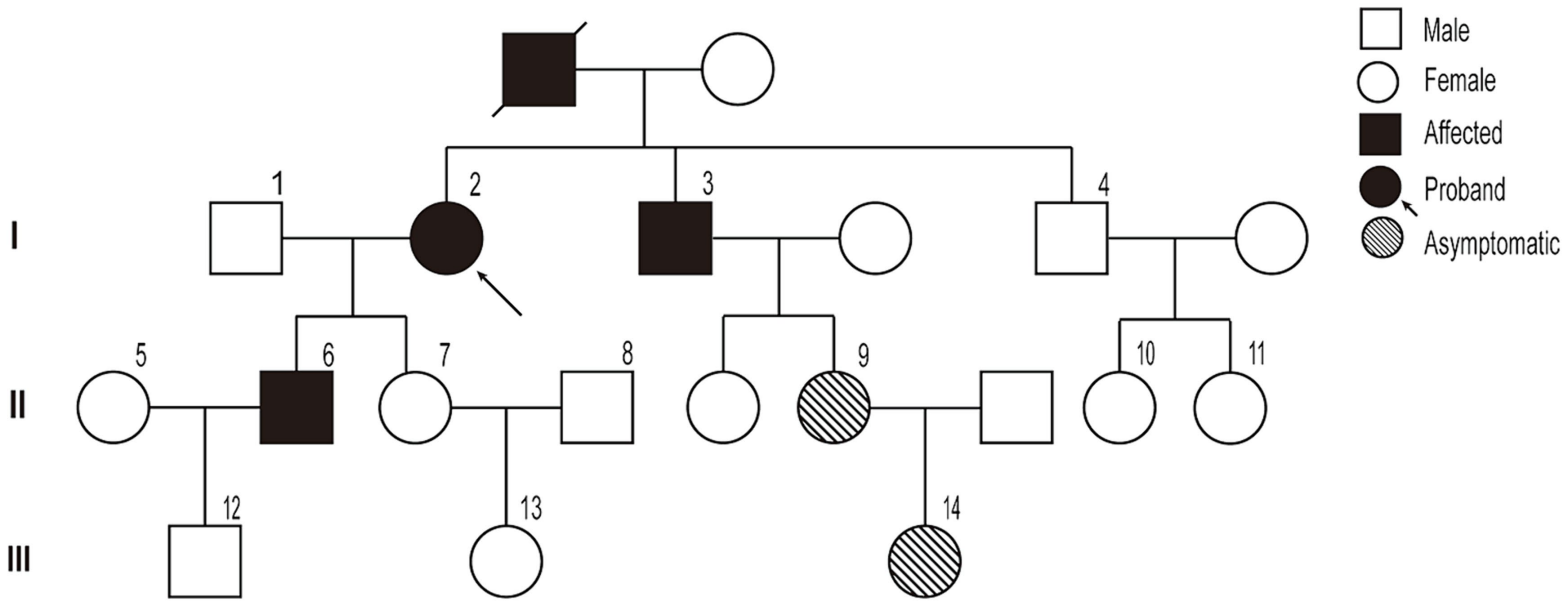
Pedigree chart of the family.

**Figure 2:**
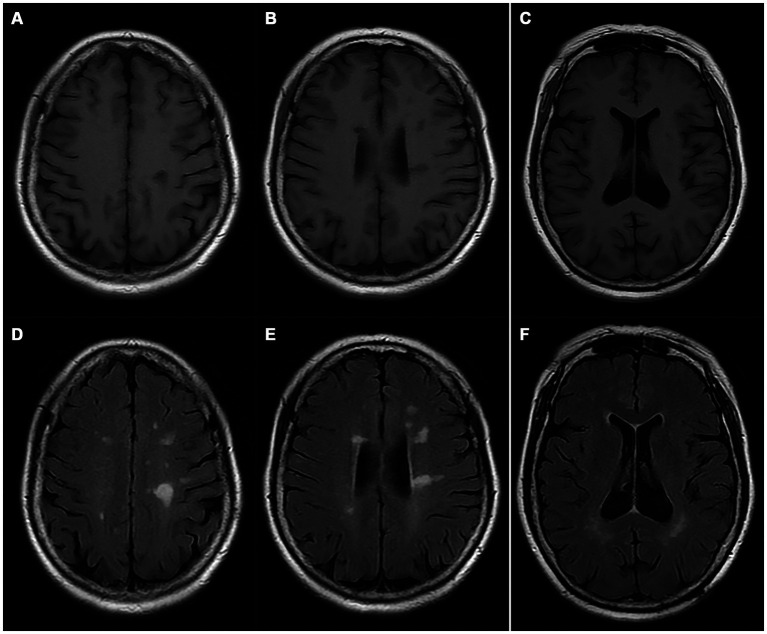
Magnetic resonance imaging of the proband (I-2). **(A–C)** T1WI; **(D–F)** T2 FLAIR images.

A pedigree chart for HSP in the family is shown in Figure 1. The clinical features of the affected individuals are summarized in Table 1, and the neuroimaging findings for the participants are shown in Figures 2A–F.

**Table 1:** Clinical features of affected individuals in the family.

Affected individual	I2	II6	I3
Gender	F	M	M
Age at onset (years)	39	36	35
Initial symptom	Spastic gait	Spastic gait	Spastic gait
Age at exam (years)	63	45	61
Gaze nystagmus	Negative	Negative	Negative
Oculomotor abnormalities	Negative	Negative	Negative
Age at loss of independent walking (IW) (years)	IW maintained	IW maintained	58
Delayed motor development	None	None	None
Intellectual disability	None	None	None
Visual system	Uninvolved	Uninvolved	Uninvolved
Dysarthria/dysphasia	None	None	None
Truncal and limb ataxia	No limb ataxia	No limb ataxia	Truncal co-ordination movement not able to access
Reflexes			
Upper limbs	+++	++	+++
Lower limbs	+++	+++	+++
Postural tremor	None	None	None
Action tremor	None	None	None
Ankle clonus	Negative	Negative	Negative
Muscle weakness or atrophy	None	None	None
Pyramidal sign or extensor plantar response	Positive	Positive	Positive
Sensory deficits	Negative	Negative	Negative
General hypotonia	None	None	None
Extrapyramidal involvement	None	None	None
Urinary/fecal urgency or incontinence	None	None	None
Peripheral neuropathy (nerve conduction studies)	Not assessed	Not assessed	Not assessed
Cerebellar atrophy	Non significance	Non significance	Non significance
MRI findings	See Figure 2	Non significance	Not assessed

Suspecting that the proband was suffering from a hereditary disease, most probably HSP, single nucleotide variant and copy number variant analyses were performed for all family members. No pathogenic copy number variants were detected in the patient. However, single nucleotide variant analysis revealed a heterozygous mutation (NM_001168272: c.2714A > G, N905S) in *ITPR1*. According to the recommendations of the American College of Medical Genetics and Genomics (17), c. 2714A > G in *ITPR1* was identified as a variant of uncertain significance (PP3 + PP3). To exclude possible genetic overlap with other nervous system diseases such as SCAs and amyotrophic lateral sclerosis (9, 18) and to further exclude dynamic mutations, large fragment deletions or duplication mutations, a panel of SCA-related gene (ATXN1, ATXN2, ATXN3, CACNA1A, ATXN7, ATXN8, ATXN10, PPP2R2B, TBP, and ATN1) testing was also conducted, using gene microarray analysis. Nothing of significance was observed.

**Figure 3:**
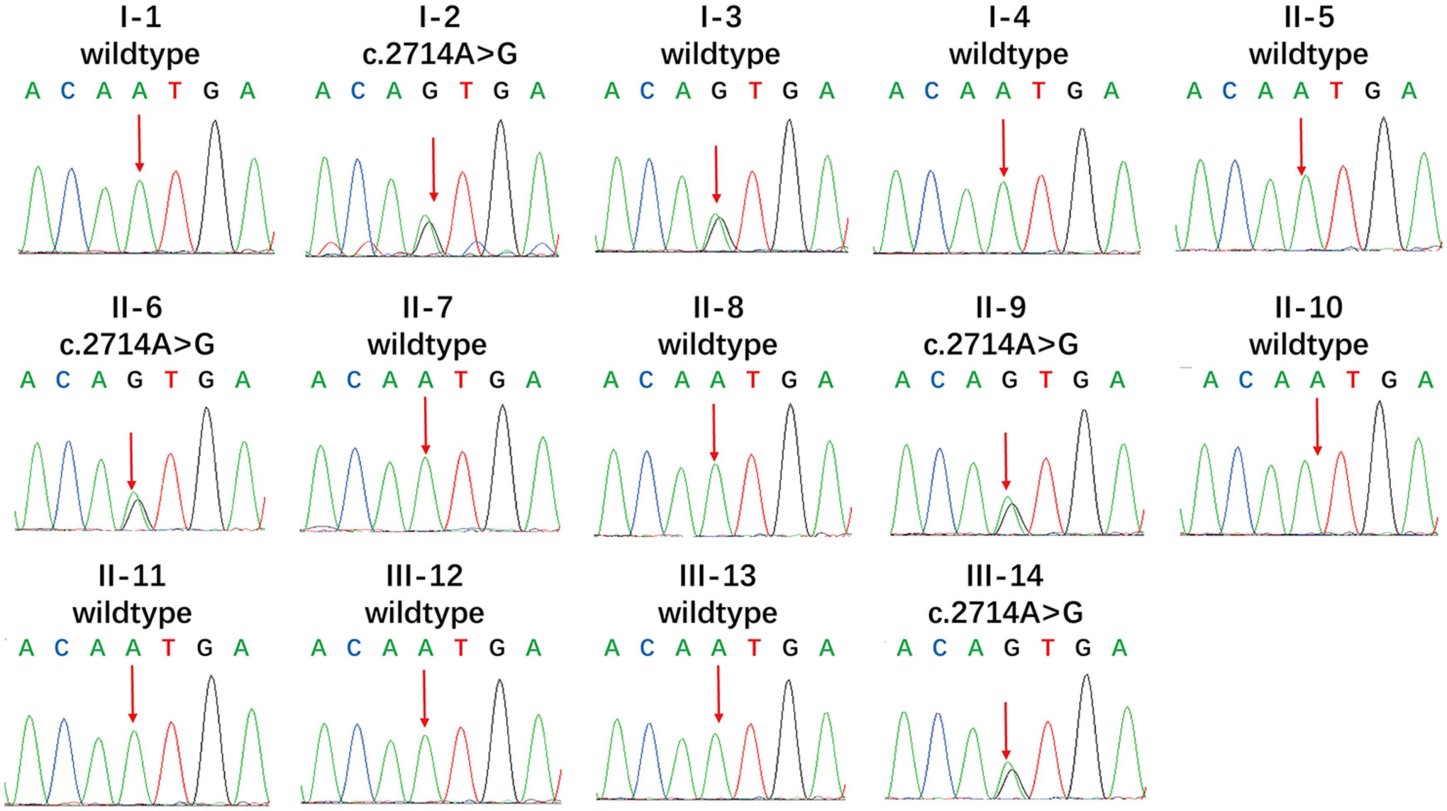
Multiple sequence alignment of mutations in *ITPR1*. Multiple sequence alignment of *ITPR1* (reference sequence: NP_001161744.1) was performed at the NCBI HomoloGene site (https://www.ncbi.nlm.nih.gov/homologene) and then aligned using DNAMAN, a multiple sequence alignment software. The results revealed specific amino acids and their conservation in other ITPR1 orthologs (across different species).

**Figure 4:**
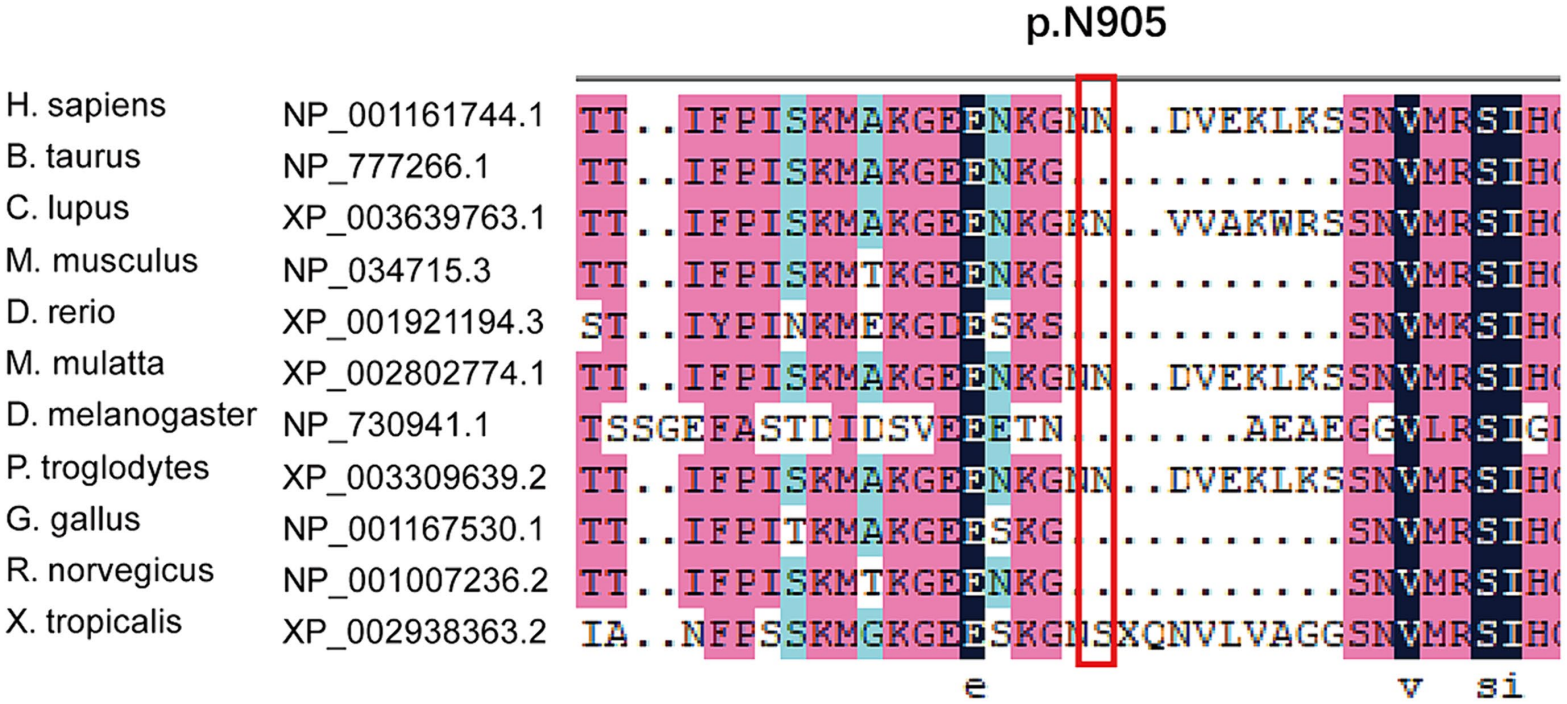
Mutational analysis of *ITPR1*. Genotypes of the proband showed a heterozygous c.2714A > G variant. Apart from the proband, I-3, II-6, II-9, and III-14 also showed the heterozygous c.2714A > G variant, while the other members showed no mutation (reference sequence: NM_ 001168272.1).

We found new variant in NM_001168272: c.2714A > G (chr3.hg19: g.4716912A > G, N905S) in *ITPR1* gene, and this missense variant locates in the same exon as the previously reported HSP-related mutation site (c.2687C > T) (9). Analysis of amino acid conservation of ITPR1 in different species showed that ITPR1 (reference sequence: NP_001161744.1) is not highly conserved in multiple species but is conserved in primates (*Homo sapiens*, *Macaca mulatta*, and *Pan troglodytes*) (Figure 3), which may indicate that ITPR1 is of structural and functional significance in primates. This variant was recognized as disease-causing by MutationTaster but was designated as benign by PolyPhen2. However, this variant was detected in all symptomatic family members and two young asymptomatic members, as confirmed by Sanger sequencing (Figure 4).

**Figure 5:**
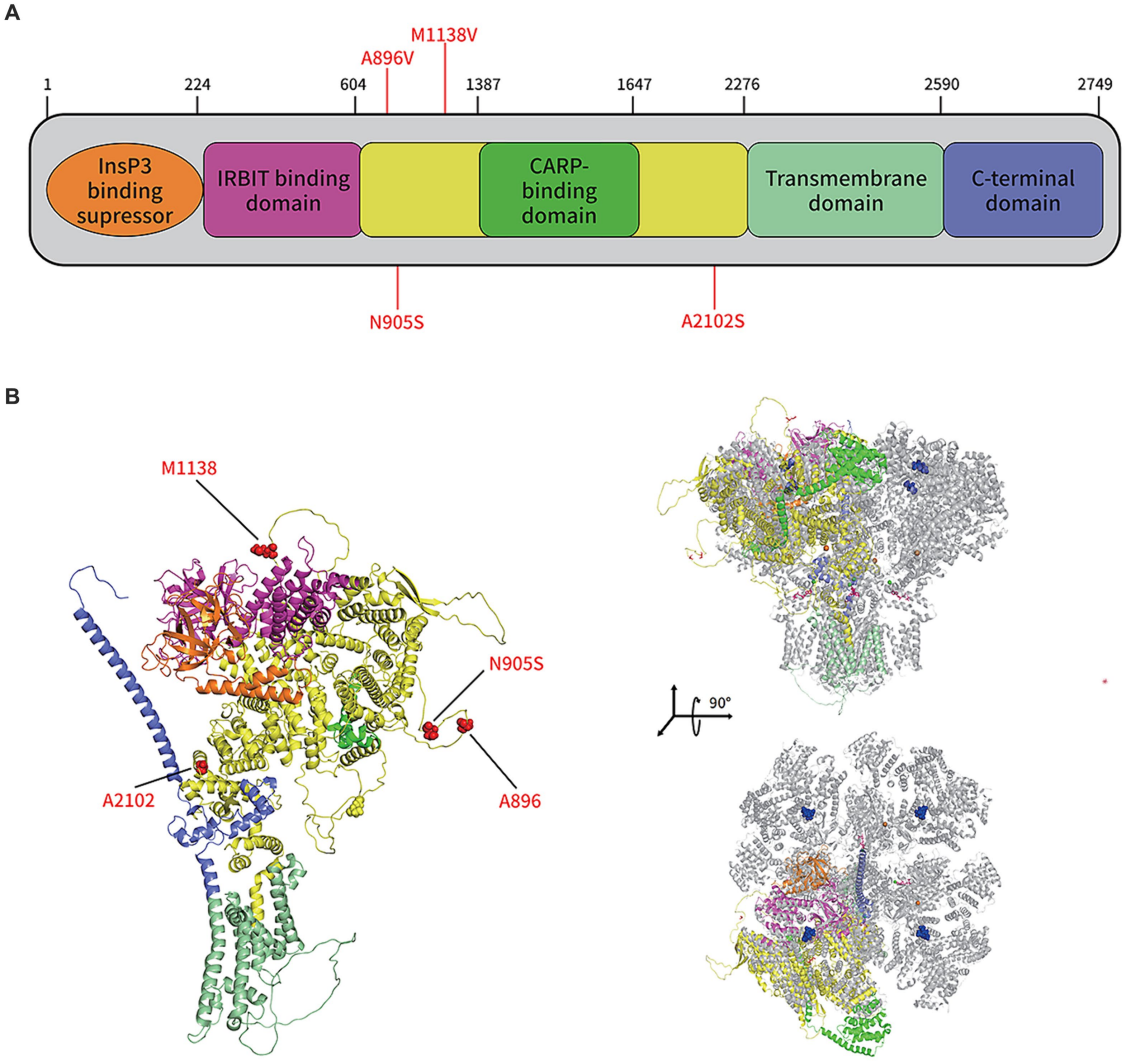
Protein structure prediction of the *de novo* mutations in *ITPR1* associated with HSP. **(A)** Schematic representation of ITPR1 (NP_001161744.1). Colored boxes represent the following domains and features: orange, Inositol 1,4,5 triphosphate binding suppressor domain; pink, Inositol triphosphate receptor-binding protein domain; yellow, coupling/regulatory domain; dark green, regulatory/carbonic anhydrase-related protein VIII binding domain; light green, transmembrane domain; and purple, C-terminal domain. Previously described mutations (A896V, M1186V, and A2102S) and the novel mutation, associated with HSP, identified in this study (N905S), are shown in red. **(B)** The structure of the ITPR1 monomer (predicted by Alphafold2, left) and structural alignment of the ITPR1 monomer with activated ITPR3 tetramers (PDB: 7T3T; blue, IP3; hot pink, ATP; orange balls, Zn2+; and green balls, Ca2+). Four mutation sites associated with HSP are shown in red.

In the protein structure analysis, the sequencing results for the patient were compared to ITPR1 (reference sequence: NP_001161744.1), which showed that asparagine at position 905 was substituted by serine. The monomeric structure of the patient’s ITPR1 was ascertained by inserting this sequence as the input for the alphafold2 software. The docking results showed that the predicted structure of ITPR1 N905S docked well with the structure of its homologous protein ITPR3, indicating the structure of ITPR1 predicted by alphafold2 was accurate. In the ITPR3 structure, amino acid residues 894–969 could not be modeled successfully because the structural details of this section are still unknown. Furthermore, as predicted by alphafold2, this section of the structure of ITPR1 N905S belonged to a loop region, and the patient’s variant site N905S was in this loop region (Figure 5).

## Discussion

In summary, we identified a new variant, NM_001168272: c.2714A > G, N905S in *ITPR1* gene from a Chinese family with several individuals affected by HSP over generations, and its transmission pattern was in accordance with the autosomal dominant pattern. The new *ITPR1* NM_001168272: c. 2714A > G, N905S variant found in this Chinese family add new evidence that this *ITPR1* mutation might be associated with HSP.

Proteins encoded by HSP genes have diverse functions, including axon transport, myelin formation, development of corticospinal tract (CST), and other neurodevelopmental processes (1). *ITPR1* is located on chromosome 3, it encodes the protein IP-3 receptor, which acts as a Ca2+ release channel and is predominantly located within the ER membrane and regulate numerous intracellular and extracellular functions via IP3 stimulation (19, 20). Typically, mutations or deletions of *ITPR1* cause SCA 15, 16, and 29 (21, 22). However, according to previous studies, different variants of this gene have varying effects on the gene and protein expression and functions. *ITPR1* is associated with hemifacial microsomia (23), ocular malformations such as the Gillespie syndrome (GLSP) (24), and ataxia (25–27), depending on the variant of its mutation, indicating the remarkable allelic heterogeneity of this gene. Mutations in *ITPR1* would not only present as a loss-of-function of the encoded protein (24) but also manifest as a dominant-negative effect (28), or gain-of-function effects, depending on the hotspots where the mutation occurs (29–31). For example, tough mutated *ITPR1* caused GLSP in humans, which typically manifests as craniofacial deformation, aniridia, and ataxia, but aniridia has never been observed in zebrafish, which possess the *ITPR1b* that is homologous to human *ITPR1* and closely related to craniofacial bone formation (23, 28, 32). Furthermore, even *ITPR1* leads to ataxia in both GLSP and SCAs; however, it only causes aniridia in GLSP, but rarely in SCAs (21, 24, 33). Therefore, *ITPR1*-related diseases have a broad spectrum of genotype–phenotype relationships. In 2019, Elert-Dobkowska et al. (9) identified three variants of the *ITPR1* mutation (c.2687C > T, c.3412A > G, and c.6304G > T) with uncertain significance in ADHSP, further expanding the phenotypic spectrum of *ITPR1*-related hereditary diseases. According to the amino acid substitutions reported for ITPR1 with an associated phenotype, (1) the IP3 binding domain, where amino acid substitutions usually cause infantile-onset SCA; (2) substitutions in the coupling domain mainly present as ataxic cerebral palsy, SCA15, and GLSP; and (3) the transmembrane domain, wherein a mutation in this domain typically will lead to GLSP and pontocerebellar hypoplasia (PCH) with ataxia. The novel NM_001168272: c.2714A > G (chr3.hg19: g.4716912A > G, N905S) is in the same exon/functional region as the variant site (c.2687C > T), which is associated with HSP and the clinical features of patients that are ITPR1-related HSP (9). Overall, the substituted amino acid encoded by the variant is in the coupling domain of the IP3 receptor, and the substitution could lead to cerebral palsy and ataxia, which gears in the phenotype of the affected individuals of the family examined in this study (34).

To date, three mutation sites (A896V, M1138V, and A2102S) on *ITPR1* have been reported to be associated with SPG (9). These three mutation sites were not involved in the same structural domain of ITPR1, as shown in the predicted structure of this protein in Figure 5, indicating high allelic heterogeneity of this gene. Furthermore, A896S is in the same loop region as the mutation N905S sequenced in this study, which implies that certain amino acid sites in this loop are closely related to SPG development. However, many researchers have noticed that most clinical genetic testing focuses almost exclusively on regions of the genome that directly encode proteins, and the disadvantages of this preference are obvious, i.e., the role of variants in non-coding regions in penetrant diseases is largely ignored (35). In a recent study, Sun et al. (36) combined WES in United Kingdom Biobank participants with imputed genotypes from FinnGen participants to conduct association meta-analyses for 744 disease endpoints across the protein-coding allelic frequency spectrum and identified 975 associations, with more than one-third being previously unreported, suggesting a gap between studies on common and rare variants. However, since the N905S variant of the patient included in this study is in an unresolved loop structure, which is flexible and conformationally variable, we were unable to determine how N905S affects the function of the protein based on the structural analysis only. Therefore, further studies are needed to investigate how this amino acid variant (N905S) contributes to the development of HSP.

Typical clinical manifestations of HSP include progressive spasms, lower limb weakness, bladder dysfunction, and a mild degree of physical dysfunction (10). The paradigmatic pathogenic involvement of HSP consists of the degeneration of CST axons and fasciculus gracilis fibers (37, 38). The pathogenic mechanisms associated with the clinical features and imaging abnormalities of HSPs vary substantially depending on the affected gene. *ITPR1* gene mutations have long been recognized to cause non-progressive cerebellar ataxia and delayed motor development, known as SCAs (especially SCA 15 and 29) and GLSP. *ITPR1*-associated cerebellar dysfunction usually becomes apparent within the first year of life, and *ITPR1*-associated SCA pathogenic involvement generally causes the degeneration of CST axons (27, 39). Therefore, delayed motor development and the absence of pyramidal dysfunction are hallmarks of SCAs. Overall, approximately 75% of the individuals with SCA 29 exhibit learning difficulties (27). However, SCA15 typically does not present with an abnormal gait until adulthood, and the affected individuals remain ambulatory for several decades after diagnosis, which is, to some extent, a condition like that of the affected individuals in this family. The affected family members in this study were devoid of delayed motor development, pyramidal signs, and intelligence deficiency, nor did they present features such as nystagmus, ataxia, postural tremor, dysarthria, and hypotonia that are generally found in SCAs or GLSP. They did not present with aniridia, or severe pontine and cerebellar hypoplasia mimicking a diagnosis of PCH or PCH supporting GLSP neither, nor did they present typical MRI findings of PCH or SCAs. Instead, all three symptomatic individuals manifested typical spastic gait, hyperactive tendon reflexes, and pyramidal signs, and ach generation of this family included patients or carriers of the mutant variant, regardless of sex, suggesting that its inheritance pattern is consistent with autosomal dominant inheritance and that this was a pure ADHSP family.

The present study had several limitations. First, given the lack of functional biological studies on other families, further research is required. Second, electromyographic studies have not been conducted in symptomatic individuals, and the comprehensive phenotype of this HSP family has not yet been thoroughly investigated. Lastly, no tests were performed on model organisms or engineered human cells to validate the pathogenesis of the mutation or to confirm the association between sequencing and phenotyping. Nevertheless, this study has expanded the mutational spectrum of *ITPR1,* and the range of genotypes associated with HSP, and has also indicated the clinical heterogeneity associated with *ITPR1*.

Overall, this study identified a novel p.N905S (c.2714A > G) variant in *ITPR1*, which is a probable pathogenic mechanism in the Chinese ADHSP family examined. Therefore, the results of this study provide important data for the genetic analysis and diagnosis of HSP. We suggest that *ITPR1* gene analysis should be included in the genetic screening panel for suspected HSP cases and that whole exome sequencing is an efficient tool for analyzing potential mutations.

## Data availability statement

The datasets presented in this article are not readily available because of ethical and privacy restrictions. Requests to access the datasets should be directed to the corresponding author/s.

## Ethics statement

The studies involving humans were approved by the Medical Ethics Committee of the Ningbo Medical Center Lihuili Hospital (Approval number: KY2020PJ051). The studies were conducted in accordance with the local legislation and institutional requirements. Written informed consent for participation in this study was provided by the participants’ legal guardians/next of kin. Written informed consent was obtained from the individual(s), and minor(s)’ legal guardian/next of kin, for the publication of any potentially identifiable images or data included in this article.

## Author contributions

RL: Data curation, Formal analysis, Writing – original draft, Investigation, Project administration. XL: Data curation, Formal analysis, Writing – original draft, Investigation. CK: Data curation, Formal analysis, Writing – review & editing. FZ: Data curation, Formal analysis, Writing – review & editing. QZ: Data curation, Formal analysis, Writing – original draft. XX: Data curation, Formal analysis, Writing – review & editing. XF: Formal analysis, Methodology, Writing – review & editing. YZ: Data curation, Formal analysis, Writing – original draft, Investigation. QH: Data curation, Formal analysis, Funding acquisition, Investigation, Methodology, Project administration, Writing – original draft.

## Footnotes

^1^http://genetics.bwh.harvard.edu

^2^http://sift.jcvi.org/

^3^http://www.mutationtaster.org/
